# What Does This Mutation Mean? The Tools and Pitfalls of Variant Interpretation in Lymphoid Malignancies

**DOI:** 10.3390/ijms19041251

**Published:** 2018-04-20

**Authors:** Yann Guillermin, Jonathan Lopez, Kaddour Chabane, Sandrine Hayette, Claire Bardel, Gilles Salles, Pierre Sujobert, Sarah Huet

**Affiliations:** 1 Centre Léon Bérard, Service d’Hématologie Clinique, 69008 Lyon, France; yannguillermin@orange.fr; 2 Hospices Civils de Lyon, Centre Hospitalier Lyon Sud, Laboratoire de Biochimie et Biologie moléculaire, 69495 Pierre-Bénite CEDEX, France; jonathan.lopez@chu-lyon.fr; 3 Cancer Research Center of Lyon, INSERM U1052 UMR CNRS 5286, Equipe labellisée LIGUE Contre le Cancer, 69008 Lyon, France; sandrine.hayette@chu-lyon.fr (H.S.); gilles.salles@chu-lyon.fr (S.G.); pierre.sujobert@chu-lyon.fr (S.P.); 4 Université de Lyon, Université Lyon 1, Faculté de Médecine et de Maïeutique Lyon Sud Charles Mérieux, 69921 Oullins CEDEX, France; 5 Hospices Civils de Lyon, Centre Hospitalier Lyon Sud, Laboratoire d’Hématologie, 69495 Pierre-Bénite CEDEX, France; kaddour.chabane@chu-lyon.fr; 6 Hospices Civils de Lyon, Service de Biostatistique—bioinformatique et plateforme de séquençage haut débit NGS-CHU Lyon, 69677 Bron CEDEX, France; claire.bardel@chu-lyon.fr; 7 Université de Lyon, Université Lyon 1, CNRS, Laboratoire de Biométrie et Biologie Evolutive UMR5558, équipe biostatistique-santé, F-69424 Lyon, France; 8 Hospices Civils de Lyon, Centre Hospitalier Lyon Sud, Service d’Hématologie Clinique, 69495 Pierre-Bénite CEDEX, France; 9 Université de Lyon, Université Lyon 1, Faculté de Pharmacie Rockefeller, 69373 Lyon CEDEX, France

**Keywords:** next-generation sequencing, lymphoid malignancies, variant interpretation

## Abstract

High throughput sequencing (HTS) is increasingly important in determining cancer diagnoses, with subsequent prognostic and therapeutic implications. The biology of cancer is becoming increasingly deciphered and it is clear that therapy needs to be individually tailored. Whilst translational research plays an important role in lymphoid malignancies, few guidelines exist to guide biologists and routine laboratories through this constantly evolving field. In this article, we review the challenges of interpreting HTS in lymphoid malignancies and provide a toolkit to interpret single nucleotide variants obtained from HTS. We define the pre-analytical issues such as sequencing DNA obtained from formalin-fixed and paraffin-embedded tissue (FFPE), the acquisition of germline DNA, or the bioinformatic pitfalls, the analytical issues encountered and how to manage them. We describe the main constitutional and cancer databases, their characteristics and limitations, with an emphasis on variant interpretation in lymphoid malignancies. Finally, we discuss the challenges of predictions that one can make using in silico or in vitro modelling, pharmacogenomic screening, and the limits of those prediction tools. This description of the current status in genomic interpretation highlights the need for new large databases and international collaboration in the lymphoma field.

## 1. Introduction

Hematological malignancies, and in particular lymphoproliferative neoplasms, are characterized by considerable heterogeneity. To ensure accurate ontological classification, one needs to integrate phenotypic information from morphology and immunophenotyping as well as genetic aspects such as cytogenetics or molecular biology. More recently, the technological breakthrough of high-throughput sequencing (HTS) has provided valuable information, which is increasingly useful in the diagnostic workflow of lymphoproliferative neoplasms. For example, mutational analysis of a panel of genes can help in the establishment of a diagnosis due to the specificity of particular mutations for a given entity (for example, the *BRAF* p.V600E mutation in hairy cell leukemia). Moreover, there is increasing evidence for a prognostic role of mutational analysis (for example, the m7-FLIPI score, which increases the value of the Follicular Lymphoma International Prognostic Index (FLIPI) clinical scoring system in follicular lymphoma (FL)) [1]. Furthermore, lymphoid malignancies have entered the personalized medicine era, where treatment is based on the mutational status of a gene. For example, the EZH2 inhibitor tazemetostat appears to have a very high activity in EZH2-mutated follicular lymphoma and patients with a tumor carrying this mutation may be preferentially treated with this new agent [2].

There are many challenges that need to be overcome to ensure the optimal use of HTS in the diagnosis of lymphoid malignancies. First, tumors are genetically heterogeneous, so the analysis of a given biopsy is not necessarily representative of the whole tumor burden. Second, the DNA can be damaged by the fixation process in formalin-fixed paraffin-embedded (FFPE) specimens, which induces artifacts in the DNA sequence that need to be distinguished from real mutations. Third, the sequencing strategy should be adapted to the clinical needs; choosing an optimal panel is a compromise between clinical, economical, and practical considerations to ensure that the clinician will obtain reliable and relevant mutational data within a reasonable time. Last but not least, the bioinformatic analysis strategy is critical; most importantly, the strategy of variant calling and filtering can introduce great variability that needs to be assessed and controlled [3]. Most laboratories perform tumor-only sequencing (i.e., without a paired germline sample), therefore it can be difficult to assess if a variant was somatically acquired during oncogenesis, and to determine its phenotypic consequences. In this review, we will describe the existing databases and strategies that can help to deal with these issues.

## 2. Is It a Somatic Variant?

The first challenges faced when dealing with HTS data from tumor tissues are to distinguish true sequence variations from technical artifacts, and then to distinguish whether a variant is somatically acquired by the tumor (or even potentially by non-tumor cells) or represents a germline variant that may or may not be implicated in tumorigenesis. To help deal with this problem, the American Society of Clinical Oncology (ASCO) and the College of American Pathologists (CAP) have published practical guidelines for the interpretation and reporting of sequence variants [4], which have been since updated twice [3,5].

### 2.1. Pre-Analytical and Bioinformatic Issues

#### 2.1.1. Obtaining Germline DNA

Ideally, one would compare tumor samples to germline DNA (e.g., from blood, saliva, hair, skin, or nails). This is sometimes difficult in routine practice due to the technical difficulties in obtaining a sufficient amount of DNA from these sources (nails, hair), invasiveness (skin), or the possible contamination by cells from the hematological lineages. Moreover, this strategy represents an additional expense.

#### 2.1.2. Technical Considerations: FFPE Tissues

FFPE tissue is the most common form of tissue that is received for molecular testing in the diagnostic laboratory. The fixation process causes various damages to DNA [6], making it challenging to interpret the variants identified by massively parallel sequencing. Formaldehyde is highly reactive with DNA bases and proteins, generating crosslinks that affect both the isolation of DNA and the amount of amplifiable DNA. Formalin fixation also causes DNA strand to break, leading to extensive fragmentation of DNA and low amounts of template amplifiable by PCR. In highly fragmented DNA samples, detecting true mutations is challenging because of the stochastic variation in allelic representation. The risk of false negatives is particularly increased if tumor purity is low [6]. Moreover, uracyl and thymine, which result from the deamination of cytosine and 5-methylcytosine by formaldehyde, are present in disproportionate levels in FFPE samples when compared to matched-frozen tissues [7]. Amplification by PCR generates C:G > T:A false-positive variants by incorporating an adenine opposite the uracil lesions. This is particularly important in the low (<10%) allele frequency range and inversely correlated with coverage, because of stochastic enrichment in the low copy number context [8]. Amplicon-based sequencing strategies are more prone to these false positive calls than capture-based approaches as they do not retain the information on the number of initiating templates of sequence reads. Accuracy and sensitivity can be improved by workflows that allow sequencing of sense and antisense strands independently (artifacts will be present in only 1 of the 2 DNA strands) [9]. Molecular tagging of the DNA templates by unique molecular identifiers (UMI) is another powerful way to reduce these FFPE artifacts (true mutations are present in all daughter molecules) [10]. Finally, variants (and in particular those of low allele frequency) should be confirmed by replicating the experiment, sequencing the antisense DNA strand, or validated by another approach. Minimizing these artifacts arising from formalin fixation is crucial to accurately detect actionable mutations from the FFPE samples. For all these reasons, if available, fresh tissue is the preferred source of DNA for molecular testing.

#### 2.1.3. Bioinformatic Aspects

There are different ways to generate a list of variants from raw HTS data: first, most sequencer manufacturers and some reagent manufacturers offer their own analysis pipelines. Second, several stand-alone or cloud-based commercial software packages are available. Third, numerous open source bioinformatic tools are shared by the scientific community. A commonly used variant caller in laboratories performing constitutional HTS is the Genome Analysis Toolkit (GATK) [11], an open source suite developed at the Broad Institute. However, variant calling of somatic samples is more complex as true mutations can often be found with a variant allele frequency (VAF) of less than 10% (see below). Consequently, laboratories performing somatic HTS use various analysis pipelines without a clear gold standard. In this context, one strategy to increase true positive results is to combine outputs from two or more variant callers (e.g., Mutect2 of the GATK suite, VarScan2, VarDict, Freebayes…) [12,13,14,15] as a combination of different callers will perform better than each variant caller taken separately [16,17,18].

Irrespective of the bioinformatic pipeline used, each variant is characterized by its VAF, which is the proportion of alternate sequences at a given position. Many confounding factors can interfere with its interpretation. In an ideal sample containing a pure tumor with only one clone, the VAF would be either 100% or 50% if the variant were homozygous or heterozygous, respectively. However, samples also contain normal cells, hence lowering the observed VAFs. Moreover, tumor cell heterogeneity will also induce a high range of mutation VAFs. This challenges the accurate interpretation of variants as true variants with a low VAF might be hard to distinguish from technical noise. Different strategies aiming to distinguish low-frequency variants from sequencing background noise have been proposed [19,20,21,22].

Another important confounding factor to consider is the copy number variation (CNV). Indeed, when a mutated gene is present in more than one copy (or when a deletion occurs on the wild-type allele), it will artificially increase the VAF leading to interpretation errors. For example, in a sample containing 66% of tumor cells that all harbor a loss of a particular genomic region, a mutation located on the remaining allele would be detected in approximately 50% of the reads (66% of cells with one single mutated allele and 34% of cells with two wild-type alleles, 66/(66 + 34 × 2) = 50%). Such a VAF of 50% is reminiscent of a germline polymorphism and can lead to a misinterpretation. Conversely, a germline polymorphism, initially present in one out of two alleles in each cell, would then be detected in 25% of the reads if the alternate allele were deleted in 66% of tumor cells (34 alleles with the variant for 34 + 66 wild-type alleles, 34/134 = 25%). Such a VAF of 25% might wrongly lead to consider a rare, germline variant as a somatic mutation. This information is not available with all sequencing techniques, therefore remains hidden in most studies. Yet, copy number alterations impact 10% of the human genome [23], and its role in cancer is probably underappreciated.

The downside of decreasing the minimal calling VAFs is that it will also increase the calling of sequencing artifacts, which need to be filtered out using different strategies:

- Some variant callers such as Freebayes or VarScan can be run in a multi-sample mode, allowing sequencing data to be obtained for all samples regarding a particular allele, if found to be mutated in one sample. From this, the minimal/mean/median frequency of reads supporting the altered sequence among all samples can be computed and inform about the background signal at this position; a VAF/median frequency ratio can be calculated, and a filtering threshold applied to select more probable true somatic mutations.
- The distribution of reference allele and alternate allele between forward and reverse sequencing strand should be similar, i.e., the ratio (reference forward reads)/(reference reverse reads) should be comparable to the ratio (alternate forward reads)/(alternate reverse reads). An Allele Strand Ratio (ASR) can be calculated, (reference F/R reads)/(alternate F/R reads); for real mutations ASR should be close to 1, conversely an ASR very distant from 1 suggests possible artifact variation.
- The occurrence of a variant in samples of the same run should be calculated, a too high recurrence would point out an artifact.
- >Over time, a local database of recurrent artifacts can be built to help remove known false positive calls.

The open source Integrative Genomics Viewer (IGV) allows visualization of the sequencing reads (bam files) [24]. Loading the data of all samples from the same run allows the evaluation of the background signal. PCR artifacts are suggested by variation in the same reads (same start, same stop) and not by others. Visualizing mutations in IGV (or a similar viewer) is essential before inclusion in a clinical report.

### 2.2. Databases for Germline and Somatic Variants

After the variant has been identified as true (i.e., not artifactual), the question of whether it is a germline polymorphism, or an acquired somatic mutation could be difficult to answer, particularly given that most labs do not perform the sequencing of germline DNA in parallel to that of the tumor. The first step to help filter out germline polymorphisms is an assessment of the VAF. Then, the phenotypic evaluation of the analyzed samples represents important information as the level of tumor purity is crucial to analyze the meaning of variants. Beyond the characterization of the sample, the interrogation of large genomic databases is required. However, one needs to understand how these databases are produced: where does the DNA come from (healthy and/or sick population, ethnic and geographical origin), how it was analyzed (quality metrics), and what kind of information is released. Various types of databases are relevant for the interpretation of somatic sequence variants including constitutional population-based databases to exclude polymorphisms and cancer/mutation-specific databases. In this article, we will focus on the databases that are most relevant and helpful when analyzing a sequence variant in routine practice in oncology.

#### 2.2.1. Constitutional Databases

These catalogues of germline variants intend to provide a comprehensive list of variants and their frequency in the general population. In a cancer analysis setting, they are useful to exclude some polymorphisms that could be interpreted as somatic (Table 1 and Table 2). Nevertheless, population-based databases should be interpreted with caution. One metric often described is the minor allele frequency (MAF), which represents the frequency at which the second most common allele occurs in a given population. As the MAF varies with ethnicity, the interpretation of a variant should include this information. Importantly, most of the databases are skewed towards an overrepresentation of the Caucasian population. Moreover, some well-established oncogenic mutations are described in these databases, which is not surprising given the high prevalence of detectable oncogenic mutations in healthy people [25]. To stress this point, we will use as an example the MYD88 p.L265P mutation, initially described as a polymorphism. This problem indeed raises epistemological questions about the causal role of gene mutations in oncogenesis, which are neither necessary [26,27] nor sufficient [28]. There is no consensus on the MAF threshold to be used to filter out potential polymorphisms, but most clinical studies use a 1% cut-off to filter-out such inherited polymorphisms.

- **The Single Nucleotide Polymorphism Database (dbSNP) of nucleotide sequence variation (SNV) from the National Cancer Bioinformatics Institute (NCBI)**: This public domain catalogue, started in 1998 [32] and first released in 2000 by the NCBI as a part of the PubMed website includes only variants from non-tumor samples. The definition of small variations comprises SNPs, small (<50 nucleotides) insertions or deletions (InDels), and retroposable element insertions and microsatellite repeat variations. The catalogue is based on voluntary contributions as any public laboratory and private organizations can submit data that, after review, will be implemented as “first class data”. The “second-class data” available are computed from the original submitted data, automatically gathered from PubMed during the dbSNP build cycle. In 2004, its false positive rate was estimated at 15–17% by Mitchell et al. [34] mainly due to its conception. It accepts submissions from many sources that are difficult to verify regarding uncritical bioinformatic alignments of highly similar but distinct DNA sequences, or PCRs with primers that cannot discriminate between similar but distinct DNA sequences. In February 2017, the last build regarding Homo Sapiens was released, build 150 comprising 325,658,303 variants of which 135,967,291 were validated (as first-class data). In this database, MYD88 L265P is known as rs387907272, and was considered as a SNP until very recently. It has now been curated as a single nucleotide variant with a pathogenic clinical significance and a variant frequency <0.01%. This well-known pathogenic mutation highlights the difficulty to filter out polymorphisms due to false positives in such databases that are constantly evolving.
- **1000 Genomes Project**: The goal of the 1000 Genomes Project was to identify genetic variants with frequencies of at least 1% in the populations studied. It ran between 2008 and 2015 and sequenced 2504 samples from 26 populations [30,31]. Whilst the samples for the 1000 Genomes Project had no associated medical or phenotype data, all participants had to declare themselves to be healthy with self-reported ethnicity and gender. MYD88 L265P is described with an overall allele frequency of 0.02%, from one non-Finnish European population. In this population, the MAF was still below a threshold that most studies would consider as rare (<0.01%) [35]. It is of note that the SNPs and short Indels of the 1000 Genomes Project are included in the dbSNP, making it redundant to consult both databases.
- **The Exome Aggregation Consortium (ExAC)**: This database, compiled by the Broad Institute, tends to aggregate and harmonize exome sequencing data from a variety of large-scale sequencing projects. All of these projects provided their raw sequencing data (generated using various technologies), which were then reprocessed and variant called through one unique pipeline to increase consistency. Notably, the germline information obtained by The Cancer Genome Atlas (see below) is available in the ExAC database.Given the nature of the projects aggregated (such as “Inflammatory Bowel Disease”, “Jackson Heart Study”, or “Schizophrenia Trios from Taiwan”), not all of the patients sequenced were healthy. In ExAC, 60,706 unrelated individuals have been sequenced [29], where people with severe pediatric diseases and their first-degree relatives have been removed. This database is not intended to be further extended, and a new project called “The Genome Aggregation Database” (gnomAD; see below) includes all the data contained in the ExAC database. In this ExAC database, MYD88 p.L265P is present at a frequency of 0.01% in the general population and is considered as too common to plausibly cause disease.
- **The Genome Aggregation Database (gnomAD)**: This database aims to aggregate the data from genome and exome studies into one database and is mainly driven by the Broad Institute. It uses the data from the ExAC database and from a consortium of more than 100 investigators and uses the same process as that used for ExAC (same pipeline and variant calling to re-process all data). It therefore contains data from the ExAC, 1000 Genomes Project, and the Cancer Genome Atlas (TCGA; see below) among others and spans 123,136 exomes and 15,496 genomes from unrelated individuals. In the gnomAD, MYD88 p.L265P is described with an allele frequency 0.0036% and count of 9.
- **National Heart, Lung and Blood Institute Grand Opportunity (NHLBI GO) Exome Sequencing Project (ESP)**: This is an on-going project bringing together US investigators aiming to discover new genes and mechanisms leading to heart disorders, lung disorders, or benign blood disorders. No cancer sequencing data are included in this database comprising 6503 samples in the ESP6500SI-V2 release. The variant calling and analysis of the data are centralized. A subset of the data was published in 2012 [33] and is therefore present in the dbSNP.

#### 2.2.2. Cancer Databases

There are no databases specific to lymphoid malignancies, and even if hematological malignancies display many genetic alterations, no dedicated project has been undertaken (Table 3). The TCGA, although less relevant for lymphoid malignancies when compared to other cancers, will be discussed given its importance.

- **The Cancer Genome Atlas (TCGA):** This project, that ended in 2017, was a collaboration between the National Cancer Institute (NCI) and National Human Genome Research Institute (NHGRI) [36]. More than 11,000 patients suffering from a total 33 types of cancer (in hematology, only acute myeloblastic leukaemia and diffuse large B cell lymphoma were targeted) were included. Cancer tissue and matched normal tissues were collected as well as clinical data, then sequenced, and registered.
- **The International Cancer Genome Consortium (ICGC):** The ICGC is a confederation of international working groups that aims to describe driver somatic mutations in more than 50 types or subtypes of cancers. Most working groups are required to sequence at least 500 samples by Whole Genome Shotgun analyses, with exceptions made for rare or very homogenous types of cancer. As this technique is not yet available everywhere for such large-scale projects, interim goals are accepted such as sequencing only the region of interest, analysis of low genome coverage of paired-end reads for rearrangements, or genotyping arrays. In addition to building this catalogue of somatic mutations, analyses of DNA methylation and RNA expression are planned. Processing the samples must be conducted according to the ICGC guidelines to ensure similar quality in the projects. Lymphoid malignancies are well represented with six different dedicated projects [37,38].
- **The Catalogue of Somatic Mutation in Cancer (COSMIC):** As is the case for dbSNP, two different types of data are present in the COSMIC. The first-class data is expert-curated, with manual input data after comprehensive review of selected genes after its submission by a group or laboratory. These genes are those presented in Census genes, a dynamic catalogue of genes that have been recognized as implicated in cancer that was initially published in Nature Reviews Cancer [40]. The second type is genome-wide screening data, uploaded from publications or imported from other databases such as the TCGA or ICGC. The uploaded data from publications imply that some false positives are included in this database through the lack of germline sample controls, simple laboratory errors, or poor-quality samples such as FFPE ones. Nevertheless, this catalogue is the most comprehensive resource for information on somatic mutations in human cancer and aims at providing somatic mutation frequencies [39].

Even if a somatic mutation is detected in a patient, its relevance in oncogenesis still needs to be determined separately. ClinVar [41] is a public archive of evidence-based data on the relationship between a variant and a phenotype. This NCBI project is still on-going with continuous submissions of germline or somatic variants. Other helpful tools include stand-alone software packages and web-based content such as the NCI Genomic Data Commons or cbioportal [42] that compiles different types of data from various projects in an attempt to facilitate the interpretation of detected variants. The latter started as a project from the Memorial Sloan Kettering Cancer Center aiming to sequence 10,000 genomes with a new hybridization capture-based HTS panel [43] and now contains 170 cancer studies from various consortia.

### 2.3. Assessing the Functional Consequences and Clinical Impact of the Variants

#### 2.3.1. In Silico Modelling

**Figure 1:**
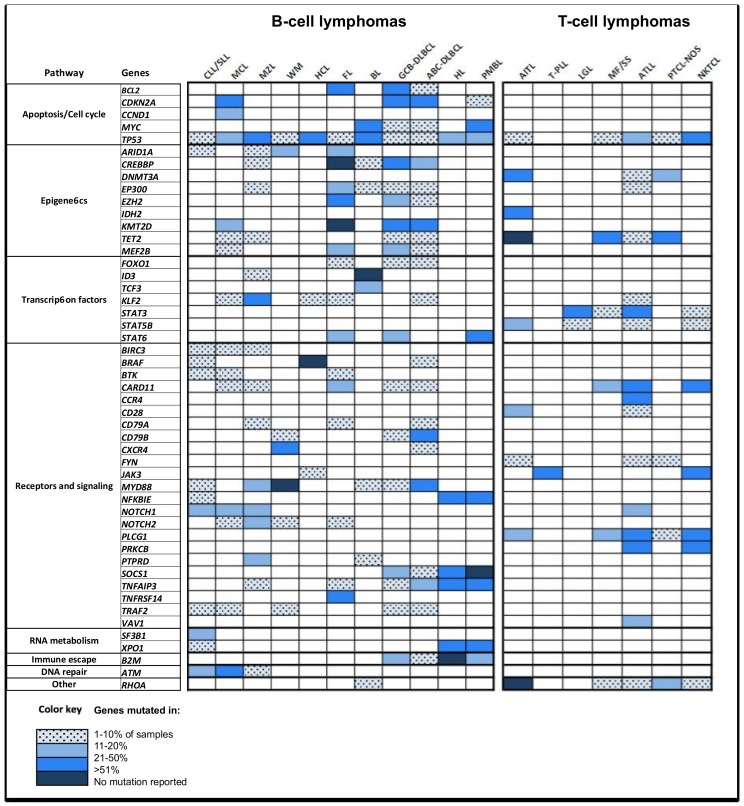
Mutation frequencies in different lymphoma entities. Abbreviations: CLL: chronic lymphocytic leukemia; SLL: small lymphocytic lymphoma; MCL: mantle cell lymphoma; MZL: marginal zone lymphoma; WM: Waldenström’s macroglobulinemia; HCL: hairy cell leukemia; FL: follicular lymphoma; BL: Burkitt lymphoma; GCB-DLBCL: germinal-center B-cell-like diffuse large B-cell lymphoma; ABC-DLBCL: activated B-cell-like diffuse large B-cell lymphoma; HL: Hodgkin lymphoma; PMBL: primary mediastinal B-cell lymphoma. AITL: angioimmunoblastic T-cell lymphoma; T-PLL: T-cell prolymphocytic leukemia; LGL: large granular lymphocytic leukemia; MF: mycosis fungoides, SS: Sézary syndrome; ATLL: adult T-cell leukemia/lymphoma; PTCL-NOS: peripheral T-cell lymphoma not otherwise specified; NKTCL: extranodal NK/T-cell lymphoma, nasal-type.

A few hotspots have been described and characterized in lymphoid malignancies (Table 4), showing direct clinical utility in refining diagnosis and/or directing treatment decisions [2,44,45,46,47,48]. The genetic landscape of most lymphomas has also been extensively studied and is now well characterized (Figure 1; for a complete review on recurrent genomic alterations, see [49,50]). Nevertheless, the functional consequences or potential clinical impact of most of the SNVs identified with HTS are currently poorly (if at all) described. In particular, tumor suppressor genes harbor a widely distributed pattern of mutations, making the interpretation of missense mutations more complex.

Several prediction tools have been developed to determine the likelihood for a variant to be deleterious (that is, to adversely impact protein function), based on sequence conservation between species, evolutionary relationship in protein family, and putative impact on the protein structure or stability [51]. A non-exhaustive list of the most widely used bioinformatic tools is presented in Table 5. These approaches, although useful, have intrinsic limitations in predicting the impact of variants on protein function. A study comparing the SIFT, PolyPhen2, and MutationAssessor algorithms in lung adenocarcinoma found a concordance of 64% between these methods, that disagreed on multiple variants in clinically relevant genes [52]. The dbNSFP aggregates functional predictions and annotations of all potential non-synonymous and splicing-site SNVs in the human genome [53,54]. The latest version (v3.5) compiles prediction scores from 20 prediction algorithms, six conservation scores, related additional annotations, and allele frequencies observed in different databases including the 1000 Genomes Project, ExAC, gnomAD, and the NHLBI GO ESP. This allows the annotation of variants and comparison of all these prediction scores in one step.

Another limitation of most databases is the lack of annotation and predicted functional impact of synonymous mutations, which change the sequence of a gene without altering the primary amino acid sequence of the encoded protein. As a consequence, they are largely ignored in clinical practice. However, accumulating experimental evidence has demonstrated that these so-called “silent” mutations actually contribute to human cancers by altering splicing, mRNA stability, protein folding, and translation [63,64]. This highlights the need to incorporate information about synonymous mutations in databases.

Deciphering whether a variant represents a driver in lymphomagenesis, and how to distinguish them from so-called “passenger” mutations is a fundamental challenge. A cancer driver mutation is defined as providing a selective growth advantage under given microenvironmental conditions, thus promoting clonal expansion. A passenger mutation provides modest (if any) advantage to the fitness of the cell, however, it might be selected in the expanding clone if it co-occurs with a driver mutation. As experimental evidence demonstrating that a mutation is responsible for a cellular phenotype that provides a selective advantage to the cell cannot be easily obtained, two approaches are considered as “surrogate” methods to distinguish driver from passenger mutations. First, the VAF has long been intuitively used as a surrogate for identifying potential drivers based on the assumption that early events, clonally represented with high VAF, likely represent alterations driving the cellular fitness and early tumor progression (although this cannot exclude a passenger mutation arising early in the progenitor cancer cells). Nevertheless, in routine practice, the VAFs are rarely corrected for copy-number status, which might greatly influence VAF. Second, several computational algorithms have been developed to address this question. Some of these in silico methods use the concept of “significantly mutated genes”, which calculate the mutation rate according to gene size and sequence context (silent mutations and non-coding mutations in the surrounding regions) to determine whether the observed mutation rate is higher than expected by chance in a given gene (e.g., MuSiC, MutSigCV) [65,66]. Other methods evaluate the expected functional impact (missense, loss-of-function, silent…) and distribution of the mutations along the gene, the entropy score for missense mutations (i.e., the degree of reoccurring mutations at a specific site within a gene), the nucleotide-level inter-species conservation, or the DNA and protein contexts (e.g., SNP density, modification of a functional domain, predicted secondary structure, change in hydrophobicity/polarity/charge…). Numerous algorithms have thus been proposed to predict the relevance of somatic mutations in cancer cells (CHASM, TUSON, Oncodrive FM, OncodriveCLUST) [67,68,69,70]. Nevertheless, a comparison showed a huge variability in the genes identified as cancer drivers by the different prediction methods, thus questioning the frequency of false-positive calls when using such algorithms [71,72]. Moreover, some of these approaches are designed for whole genome or whole exome data and are not appropriate for gene panels that are currently used in clinical practice.

#### 2.3.2. In Vitro Modelling

Beyond in silico predictions, the consequences of a given mutation can be tested in vitro. Whereas establishing an accurate model is time consuming and not compatible with the time constraints of the clinical setting, some public databases assessing the functional consequences of a large number of mutations can be very informative. However, one should keep in mind that in vitro assessment may not reproduce in vivo behavior; for example, despite all the models showing activation of the Mitogen-Activated Protein Kinase (MAPK) pathway by BRAF p.V600E mutations, colorectal cancers with this mutation are not sensitive to vemurafenib, because of the activation of a feedback loop leading to Epidermal Growth Factor Receptor (EGFR) activation [73].

• Large scale pharmacogenomic screening of cell lines:

Two major initiatives have analyzed how the sensitivity of cell lines towards a large panel of chemical compounds is correlated with genomic features. Importantly, there is a good agreement between the results obtained with these two large scale projects [74].

The Genomics of Drug Sensitivity in Cancer project has described the sensitivity of 1001 cancer cell lines to 265 anticancer drugs, and compared the response rates with the analysis of DNA sequence, copy number anomalies, DNA methylation, and gene expression at the mRNA level [75]. The data are easily accessible via a website [76], allowing the evaluation of drug sensitivity when a given gene is mutated, amplified, or deleted. However, no distinction is made between the different mutations existing for a given gene (such as gain-of-function or loss-of-function mutations). This point is of importance when interpreting these data.

The Cancer Cell Line Encyclopedia project has produced similar data [77] on 242 cell lines exposed to 354 small molecules, which was later extended to 860 cell lines and 481 compounds in an updated version. The data are also available through a web interface [78], allowing the evaluation of how genomic or transcriptomic features (mutations, CNV analysis, gene expression) impact drug sensitivity. For example, the mTORC1 inhibitor sirolimus is predicted to be more active in the case of *PTEN* mutation or deletion, as expected, and less active in the case of *CD79B* mutation. Whether these data will translate into clinically meaningful information remains to be demonstrated. Caution is required when interpreting these data, which should be considered for generation of hypotheses and not to guide patient treatment.

• Large-scale phenotypic characterization of mutations:

Recently, a team from the Broad Institute has succeeded in moving from high throughput genomic characterization of lung cancer to large-scale functional analysis of variants [52]. For the 194 most frequent mutations described in lung cancer, they measured how much the overexpression of the mutated gene impacted the transcriptome (using a reduced transcriptome called L1000) [79] when compared with the overexpression of the wild-type form; they demonstrated that only 69% of these variants had measurable functional consequences. However, to the best of our knowledge, no such database exists for lymphoma mutations, but this approach may not be feasible owing to the extreme heterogeneity between lymphoma subtypes.

#### 2.3.3. Limits of Current Prediction Tools and Models to Predict the Clinical Impact

Despite efficient prediction tools and large-scale in vitro screening, none of these approaches can totally unravel the in vivo complexity of tumor biology. A first limit is that very small mutated sub-clones might not necessarily be identified as potential drivers, and their selection under treatment is not predictable by using only in silico approaches. In chronic lymphocytic leukemia, sub-clones with *TP53* mutations have been shown to expand to dominant clones under the selective pressure of chemotherapy [80,81,82,83], and to greatly influence the response to chemotherapy and clinical outcome, irrespective of the VAF (however this concept has been challenged in a recently published study [84]). Second, the selection of small clones by a dysregulated tumor microenvironment during the course of the disease should probably reduce the importance that is given to the VAF or in silico approaches in mutational status interpretation at the time of diagnosis [80,85]. Adding another layer of complexity, the influence of a mutated clone on non-mutated neighboring cells probably represents a still unrecognized pathophysiological mechanism of drug resistance in some tumors bearing particular small mutated clones. Such a paracrine pro-survival signal was recently shown for the tyrosine-protein kinase BTK p.C481S mutation-bearing cells in Waldenström’s Macroglobulinemia (WM) and diffuse large B-cell lymphoma of the activated B-cell subtype (DLBCL-ABC) [86]. Large-scale pharmacogenomics or functional screening of cell lines do not investigate the potential impact of the tumor microenvironment on mutated sub-clones, nor the interplay between tumor cells with different molecular features.

All of these findings confirm that limiting the tumor genetics to the dominant clone may hamper the accurate prediction of outcome and optimal therapeutic decisions. This may have direct consequences for the design of clinical trials when patient enrolment relies on mutational status, for example, whether a VAF threshold should be applied to enroll patients for targeted therapy, and whether a tumor with a small mutated sub-clone will respond similarly to one with a predominantly mutated population. Recent data suggest that the presence of small BTK p.C481S mutation-bearing clones may determine the response to BTK inhibitor ibrutinib [86].

The combined effect of co-occurring mutations might also greatly influence the biological and molecular properties of tumors, and the response to treatment. Two striking examples of such interaction were recently described in B-cell lymphomas: in WM, patients with MYD88^L265P^ and CXCR4^WT^ status showed the highest response rate to ibrutinib when compared to MYD88^L265P^CXCR4^MUT^ and MYD88^WT^CXCR4^WT^ patients [46]; in DLBCL, tumors with CD79A/B^WT^MYD88^MUT^ did not respond to ibrutinib, whereas CD79A/B ^MUT^MYD88^MUT^, CD79A/B^WT^MYD88^WT^, and CD79A/B^MUT^MYD88^WT^ tumors did [44]. Nevertheless, in silico prediction of the impact of associated variants is highly challenging. To the best of our knowledge, this issue was addressed by at least one publication [87], but evidence regarding the accuracy of those predictions is lacking. Finally, the order in which the associated mutations were acquired in tumor-initiating cells has been shown to influence clinical features and the response to targeted therapy in myeloproliferative neoplasms [88,89], but such evidence has yet to be demonstrated in lymphoid neoplasms. Future studies incorporating single-cell genotyping might answer this question in lymphoma.

## 3. Conclusions

HTS allows refinement of molecular diagnosis in lymphoid malignancies, which has therapeutic and prognostic implications. For these reasons, it is becoming a method of choice for variant detection by many clinical laboratories. However, the difficulties for an accurate and reproducible report of variants between platforms and laboratories are far from trivial and span technical, computational, and biological challenges in data interpretation. For the assessment of the functional consequences of variants identified in lymphoid malignancies, specific databases, or the extension of existing ones are needed. Such a database dedicated to lymphoproliferative disorders could be built by collecting all known gene lesions published in peer-reviewed literature, and/or from user-submitted data, followed by manual curation on an ongoing basis. Given the expansion of the HTS studies, a large number of lymphoid variants might be quickly listed in a specific database that would be further implemented for both novel mutations and additional annotations. Ideally, the information needed would include the validation in germline controls (i.e., whether a mutation was proven to be somatic), functional consequences from in vitro experiments (if assessed), correlation with other genomic, epigenomic or transcriptomic features of the tumors, phenotypic impact on drug sensitivity (if assessed), and curated information regarding the lymphoma subtype (such as detailed histology report) to allow appropriate interpretation.

## Figures and Tables

**Table 1:** Catalogues of germline variants databases.

Database	Cell of Origin	Healthy/Non Cancer Disease	Data	Number of Exome/Genome	URL
ExAC	Germline	both	Exome	60,706	http://exac.broadinstitute.org/
gnomAD	Germline	both	Exome/Genome	136,632 *	http://gnomad.broadinstitute.org/about
1000 Genomes	Germline	Healthy	Exome/Genome	2504	http://www.internationalgenome.org/home
dbSNP	Germline	both	Exome/Genome	NA	https://www-ncbi-nlm-nih-gov.gate2.inist.fr/projects/SNP/
ESP	Germline	both	Exome	6503	http://evs.gs.washington.edu/EVS/

* 123,136 exomes and 15,496 genomes; NA: No data available.

**Table 2:** Ethnic representation in each database of germline variants, and reported frequencies for the MYD88 p.L265P mutation.

Database	% African/African American	% Latino/Mixed Americans	% East Asian	% Finnish	% Non-Finnish European	% South Asian	% Ashkenazi	% Other	MYD88 p.L265P Allele Frequency	Reference
ExAC	8.57	9.53	7.13	5.45	54.97	13.6	NA	0.75	0.01%	[29]
gnomAD	8.80	12.60	6.91	9.44	46.38	11.26	3.72	2.37	0.0036%	[29]
1000 Genomes	26.4	13.86	19.53	3.95	16.13	20.13	NA	0	0.02%	[30,31]
dbSNP	NA	NA	NA	NA	NA	NA	NA	NA	*	[32]
ESP	NA	NA	NA	NA	NA	NA	NA	NA	Not present	[33]

* dbSNP reports VAF from different studies; NA: No data available.

**Table 3:** Catalogues of cancer databases.

Database	Cell of Origin	Data	Number of Exome/Genome	Link	Reference
TCGA	Somatic	Exome/Genome	11,077	https://tcga-data.nci.nih.gov/docs/publications/tcga/	[36]
ICGC	Somatic	Exome/Genome	17,000	http://icgc.org/	[37,38]
COSMIC	Somatic	Exome/Genome	32,000 genomes + 25,000 peer reviewed papers (genomes and/or exomes)	http://cancer.sanger.ac.uk/cosmic	[39,40]

**Table 4:** Mutation hotspots in lymphoid neoplasms.

Gene	Hotspot Mutation	Lymphoid Neoplasms (Frequency)	Commentary
*BRAF*	V600E	HCL (>90%), MM (5%)	targeted therapy available
*EZH2*	Y646, A692 *	FL (30%), DLBCL (10%)	targeted therapy available;* Amino-acid numbering based on transcript NM_004456.4 (sometimes reported as Y641 and A687 with NM_001203247.1)
*IDH2*	R172	AITL (40%)	targeted therapy available
*K/N/H-RAS*	G12, G13, Q61	MM (40%), DLBCL (10%)	targeted therapy available
*MYD88*	L265P **	LPL (95%), MGUS (50%), DLBCL (10%), CLL (5%), PCNSL (50%), EMZL/MALT (5%), NMZL (5%)	** Amino-acid numbering based on transcript NM_002468.4 (sometimes reported as L273P with NM_001172567.1)
*RHOA*	G17V	AITL (60%), PTCL-NOS (20%)	-
*SF3B1*	K700E, K666	CLL (15%)	Prognostic impact in CLL
*XPO1*	E571	PMBL (25%), cHL (25%), CLL (5%)	targeted therapy available

Only recurrent mutations observed with a frequency >10% are presented. Abbreviations: HCL: hairy cell leukemia; MM: multiple myeloma; FL: follicular lymphoma; DLBCL: diffuse large B-cell lymphoma; AITL: angio-immunoblastic T-cell lymphoma; LPL: lymphoplasmacytic lymphoma; MGUS: monoclonal gammapathy of undetermined significance; CLL: chronic lymphocytic leukemia; PCNSL: primary central nervous system lymphoma; EMZL/MALT: extranodal marginal zone lymphoma of mucosa-associated lymphoid tissue; NMZL: nodal marginal zone lymphoma; PTCL-NOS: peripheral T-cell lymphoma, not otherwise specified; PMBL: primary mediastinal B-cell lymphoma; cHL: classical Hodgkin lymphoma. * Amino-acid numbering based on transcript NM_004456.4 (sometimes reported as Y641 and A687 with NM_001203247.1). ** Amino-acid numbering based on transcript NM_002468.4 (sometimes reported as L273P with NM_001172567.1).

**Table 5:** Bioinformatic resources for prediction of variant functional impact.

Resource	URL	References
SIFTSorting Intolerant From Tolerant	http://sift.jcvi.org	[55,56]
PROVEANProtein Variation Effect Analyzer	http://provean.jcvi.org/index.php	[57]
PolyPhen-2Polymorphism Phenotyping v2	http://genetics.bwh.harvard.edu/pph2	[58]
MutationAssessor	http://mutationassessor.org	[59]
MutationTaster	http://www.mutationtaster.org/	[60]
CAROL *Combined Annotation scoRing toOL	http://www.sanger.ac.uk/science/tools/carol	[61]
Align GCGD **	http://agvgd.hci.utah.edu/	[62]
dbNSFP v3.0 ***database for Nonsynonymous SNPs’ Functional Predictions	https://sites.google.com/site/jpopgen/dbNSFP	[54]

* Combines SIFT and PolyPhen-2; ** Cancer-specific database where users can either supply their own protein multiple sequence alignments or select from the library of alignments (currently available for ATM, BRCA1, BRCA2, CHEK2, and TP53); *** compiles prediction scores from 20 prediction algorithms (SIFT, Polyphen2-HDIV, Polyphen2-HVAR, LRT, MutationTaster2, MutationAssessor, FATHMM, MetaSVM, MetaLR, CADD, VEST3, PROVEAN, FATHMM-MKL coding, fitCons, DANN, GenoCanyon, Eigen coding, Eigen-PC, M-CAP, REVEL, MutPred) and 6 conservation scores (PhyloP × 2, phastCons × 2, GERP++ and SiPhy).
